# Environment and Host-Genetic Determinants in Early Development of Allergic Asthma: Contribution of Fungi

**DOI:** 10.3389/fimmu.2019.02696

**Published:** 2019-11-20

**Authors:** Sabelo Hadebe, Frank Brombacher

**Affiliations:** ^1^Division of Immunology and South African Medical Research Council (SAMRC) Immunology of Infectious Diseases, Department of Pathology, Faculty of Health Sciences, University of Cape Town, Cape Town, South Africa; ^2^International Centre for Genetic Engineering and Biotechnology (ICGEB), Cape Town, South Africa; ^3^Division of Immunology, Faculty of Health Sciences, Institute of Infectious Diseases and Molecular Medicine (IDM), University of Cape Town, Cape Town, South Africa; ^4^Faculty of Health Sciences, Wellcome Centre for Infectious Diseases Research in Africa (CIDRI-Africa), Institute of Infectious Diseases and Molecular Medicine (IDM), University of Cape Town, Cape Town, South Africa

**Keywords:** allergy, severe asthma associated with fungal sensitivity, fungi, Low- and lower-middle-income countries, hyperreactivity

## Abstract

Asthma is a chronic debilitating airway disease affecting millions of people worldwide. Although largely thought to be a disease of the first world, it is now clear that it is on the rise in many middle- and lower-income countries. The disease is complex, and its etiology is poorly understood, which explains failure of most treatment strategies. We know that in children, asthma is closely linked to poor lung function in the first 3-years of life, when the lung is still undergoing post-natal alveolarization phase. Epidemiological studies also suggest that environmental factors around that age do play a critical part in the establishment of early wheezing which persists until adulthood. Some of the factors that contribute to early development of asthma in children in Western world are clear, however, in low- to middle-income countries this is likely to differ significantly. The contribution of fungal species in the development of allergic diseases is known in adults and in experimental models. However, it is unclear whether early exposure during perinatal or post-natal lung development influences a protective or promotes allergic asthma. Host immune cells and responses will play a crucial part in early development of allergic asthma. How immune cells and their receptors may recognize fungi and promote allergic asthma or protect by tolerance among other immune mechanisms is not fully understood in this early lung development stage. The aim of this review is to discuss what fungal species are present during early exposure as well as their contribution to the development of allergic responses. We also discuss how the host has evolved to promote tolerance to limit hyper-responsiveness to innocuous fungi, and how host evasion by fungi during early development consequentially results in allergic diseases.

## Introduction

Asthma has largely been thought to be a genetic pre-disposition, however, over the last two decades it is becoming more clear that environment may equally play a significant role in the development of the disease (1). Genetic pre-disposition is supported by numerous epidemiological data showing that children born to both parents with asthma or low forced expiratory volume per second (FEV_1_) are at higher risk of developing asthma (2). Many studies have also linked specific gene loci or single nucleotide polymorphisms (SNPs) to asthma disease in different parts of the world (3). For example, HLA-DQ, SMAD3, IL-2RB1, IL-33, IL-1RL1, IL-4RA, ADAM33, FOXP3, STAT6, and 17q21 loci, have been linked to asthma diseases in diverse ethnic groups with varying degrees (3–5). What is intriguing about asthma is that it is mainly prevalent amongst urban dwellers compared to rural areas in different regions of the world. Although asthma has always been associated with first world countries, it is clear that it is on the rise in middle to lower income countries and in some of these countries the incidence rates may be as high as Western world countries (6, 7). This points to additional factors that can contribute to the rise in asthma disease, apart from genetic pre-disposition. Indeed, environmental factors are now considered as another possible factor that contributes to the increase in asthma. This stems from many epidemiological data mainly from North America, Scandinavian and Eastern European countries that have demonstrated that children raised in farm environments tend to be protected from asthma, whereas those from urban “cleaner” environments are at higher risks (8–10). A term now widely known as “hygiene hypothesis” coined by David Strachan in the late 80s (10–12). The hygiene hypothesis is primarily based on exposure to microbes or high endotoxin levels in the environment in early life which leads to reduced allergic asthma. Hygiene hypothesis may not fully explain the rise of asthma as some studies have disproved this early exposure to high endotoxins and reduced asthma risks (13, 14). A study by Litonjua et al. (15) in children under the age of 5 years exposed to high concentrations of endotoxin in house dust mites were at high risk of developing wheeze over a 46 months period. Other studies have also found variable influence of endotoxin levels in asthma and atopy development in early life depending on dose, exposure and timing (16). Apart from endotoxin level studies, other studies focusing on Bacilli Calmetté-Guerin vaccination and early *Mycobacterium tuberculosis* infection amongst children in both high- and low-income countries, could not establish an association between infection or immunization with allergen sensitization (17, 18). Other studies have also shown no association between previous infection with *Toxoplasma gondii*, herpes simplex virus, hepatitis A or *Helicobacter pyroli* with allergen sensitization and allergic diseases amongst Spanish and German university students (19). Other factors linked to increase in asthma are the rise of industrialization in many countries which generates large amounts of air pollution. *In utero* pregnant mothers and early life exposure to tobacco smoke is linked to poor lung function and development of chronic obstructive pulmonary disease (COPD) and early mortality (2, 20). Thus, the relationship between early life exposure to microbes or air pollutants and asthma is complex and is likely to be dependent on many factors, such as the nature of exposure, how and when it takes place, host genetic susceptibility and probably many other factors currently not defined (21).

All these do point to the environment being important in asthma pathogenesis. However, other microbes such as fungi have been linked to exacerbations of asthma in both children and adults, suggestive of a non-protective role (22, 23). Naturally, fungi is not detected in high levels in healthy human lung, which had lead researchers to assume that lungs contain no fungus. This is partly due to the fact that inhaled fungal spores are rapidly cleared by both active physical forces such as mucociliary lining the lung epithelium and clearance by innate immune phagocytic cells, such as macrophages. The lack of highly sensitive techniques to detect fungi in sputum or lavage fluid and cumbersome techniques to detect species, partly contributes to low detection of fungi in healthy human lung (24). Nevertheless, some studies have detected certain phyla in healthy respiratory airways including fungi belonging to the genera *Cladosporium, Eurotium, Penicillium*, and *Aspergillus*. Other genera such as *Candida, Malassezia*, and *Pneumocystis* have been detected, but in low abundance (25). It is likely that some of these species of fungi found in lung sampling may also be part of the oral mycobiome, as techniques used to sample lavage fluid or endoscopy can be contaminated with oral cavity fungi. Some fungi are often detected in sputum or lavage fluid in chronic respiratory diseases such as asthma. These fungal species exploit a compromised host defense system, often germinating, and colonizing the airways, causing mycosis which precedes sensitization and exacerbation of asthma symptoms (26, 27). Some of the fungal species associated with asthma are shown in Table 1. These include genera *Aspergillus, Candida, Alternaria, Cladosporium*, and *Cryptococcus* spp. amongst others (35, 40). Some of these species isolated from airways or sinusitis of asthmatic patients with TH2-type disease show viability when cultured *in vitro*, showing that infection is essential in disease manifestation (27). Importantly, these patients generally show increased fungal-specific IgE responses, memory phenotype when stimulated with fungal antigens and eosinophilia which is directly involved in fungal killing (28, 41). It is now clear that fungal colonization contributes largely to asthma and its severity, however, current methods used to determine colonization rely on culture methods for viable fungi and molecular methods which do not necessarily differentiate between active colonization and fungal fragments. This leads to underestimation of disease burden and bias in distinction between disease and colonization depending on detection methodology used. The mode of sensitization is mainly aerosol exposure, however, some fungal species are commonly found colonizing the skin, for example *Malassezia* spp. and *Trichophyton* spp. This suggests that there might have a different mode of sensitization when found on the skin which leads to asthma. These fungal species, although generally found colonizing the skin have also been detected in the both healthy and asthmatic lung and gut which may suggests diverse adaptation in multiple barrier sites (35). In this review, we mainly focus on how some fungi may cause or contribute to allergic asthma, we also discuss how fungi such as *Malassezia* and *Trichophyton* that mainly colonize the skin can be important sources of allergens that sensitizes the skin and cause allergic asthma through atopic march (42). We discuss some of the genetic risk factors associated with fungal asthma and tolerance mechanism that host has developed to limit hyperreactivity to these ubiquitous organisms.

**Table 1:** Some of the fungal species associated with allergic asthma via various routes of sensitization.

**Fungal genera**	**Fungal species**	**Allergens**	**Activity**	**Route of exposure**	**Reference**
*Aspergillus* spp.	*A. fumigatus* *A. nudilans* *A. versicolor* *A. flavus*	Asp f 1 Asp f 3 Asp f 11, Asp f 27 Asp f 22	Ribotoxin Perioxisomal membrane protein Protein folding Enolase	Inhalation	(27–29)
*Alternaria* spp.	*A. alternata*	Alta 1 Alta 2 Alta 4	Transporter, disarms defense Protein initiation factor Thioredoxin	Inhalation	(22, 24, 30, 31)
*Cladosporium* spp.	*C. clasosporioides* *C. herbarum*	Clac c9 Clac c14 Clac h8	Peroxysomal protein Transaldolase Mannitol dehydrogenase	Inhalation	(32–34)
*Malassezia* spp.	*M. globosa* *M. furfur* *M. sympodialis* *M. restricta* *M. japonica*	MGL_1304 Mala f 2-4 Mala s 1-11, MalaEX	Not known Stress responses Protease activity Vesicle trafficking and innate immune responses	Skin contact Inhalation Ingestion	(35, 36)
*Cryptococcus* spp.	*C. neoformans* *C. gattii*	Chitin Chitosan Mpr1	Immune activation CNS invasion	Inhalation	(37, 38)
*Trichophyton* spp.	*T. interdigitale* *T. tonsurans* *T. mentagrophytes*	Trit 1 Trit 4	Enzymatic activity? Sporulation?	Skin contact	(39)

## Fungal Sensitization and Asthma Risks

Sensitization to fungi poses a major risk factor for asthma including severe form of asthma and death. Sensitization to fungi is estimated anywhere between 2 and 90% in asthmatics (24, 43). Factors that affects this variation include different exposure, batch of commercially available skin prick test (SPT) extracts and subjective methods used to measure exposure. There is also a high degree of multiple fungal species sensitization, which further complicates these estimates. For example a study done in multiple states within the United States of America using a Pharmacia Capsulated hydrophobic carrier polymer (CAP) system, did show a positive correlation between sensitization to outdoor *Alternaria* and indoor *Cladosporium* (44). Four fungal genera namely, *Alternaria, Aspergillus, Penicillium*, and *Cladosporium* are overly represented when it comes to measuring fungal allergen exposure (43, 45, 46). This is partly due to airborne abundance, distinct morphological characteristics, geographic locations, and whether indoor or outdoor allergens are being considered.

Highest asthma exacerbations are observed during sporulation seasons, however, most individuals are exposed to either sub-micron (>1 μm) or larger (<1 μm) fungal fragments (23, 43, 47). These fungal fragments are derived from broken or fractured conidia or hyphae and can be easily dislodged by penetrating deeper pockets of the lung at much higher concentrations than spores or conidia (46, 48, 49). This is particularly an efficient aerosolization model for some of the larger fungi such as *Alternaria alternata* that can be over 10 μm in size and can only penetrate upper respiratory airways (48, 50). Determining whether an individual patient has been sensitized through fungal fragments or live infection is difficult to ascertain without a positive culture, or biopsy for histopathology. However, several case reports have shown that fungal mucosal infection precludes sensitization to that fungus and exacerbates life-threatening allergic asthma (27, 51, 52). Treatment with antifungal drugs and low dose corticosteroids improves asthma symptoms and clears fungal infection, suggesting that airway mycosis may be important in subsequent sensitization (23, 51, 53). It is likely that infectious mycosis instigates sensitization through activation of TH2-type immune response, while fungal fragments derived from hyphae and conidia can exacerbate allergic asthma through their easily accessible fungal pathogen associated molecular patterns (PAMPs) (46, 49). Several animal model studies also support the idea that airway mycosis induces TH2-type airway disease and that some fungal PAMPs may exacerbate allergic asthma in the presence of allergens such as ovalbumin or house dust mite (28, 54–56).

Some studies have suggested that infants are likely to be exposed more than adults and more fungal fragments seem to seed deeper pockets of the lung, which may explain increased risk to fungal induced asthma (49, 57). However, variation in exposure and allergic asthma has also been observed in children from low-income and high-income homes, which may suggests that exposure is also biased based how often one visits a physician. Children from high income homes are likely to be diagnosed with fungal induced asthma simple because of access to healthcare, whereas children from low income homes may even have higher exposure due to living in damp, moldy homes, with less access to healthcare and diagnosis (58).

## Genetic Basis for Fungal Induced Asthma

Asthma is a multifactorial complex disease whose origins and pathogenesis are still not clear. Multiple genetic risk factors involved in inflammatory pathway of allergic asthma have been reported. These polymorphisms associated with asthma risk are not present in all patients and seem to be influenced largely by environment. Interleukin 4 receptor alpha (IL-4Rα) and IL-13 polymorphisms are associated with hyper IgE and asthma severity (59, 60). There are currently eight known naturally occurring SNPs of the IL-4RA namely ile75val, glu400ala, cys431arg, ser436leu, ser503pro, gln576arg, ser752ala, and ser786pro (61, 62). Some of these SNPs are associated with *A. alternata* moderate to severe asthma in children (63). HLA-DQB1^*^03 and HLA-DRB1^*^13 have also been associated with *A. alternata* moderate to severe asthma in children, where HLA-DQB1^*^03 was reduced in frequency and HLA-DRB1^*^13 increased in frequency when compared to *A. alternata* mild asthmatic children (63). These SNPs were specific to fungal sensitization as they were not present in control Allergic Bronchopulmonary Aspergillosis (ABPA) or Chronic Pulmonary Aspergillosis (CPA). Other studies have shown susceptibility variants specific to innate immune responses and pattern recognition. Toll-like receptor 3(TLR-3), TLR-9, DECTIN-1 variants were shown to be associated with severe asthma with fungal sensitization (SAFS) induced by *Aspergillus fumigatus* in a Caucasian older cohort (64, 65). In another study, DECTIN-1 variant rs58677678 was found to be associated with reduced gene expression leading to unrestrained type 2 immune response and subsequently poor lung function (66). Other SNPs associated with SAFS in this cohort were genes associated with TH2 chemoattractant (CCL17) and monocyte recruitment (CCL2) or immune suppression (IL-10). Interestingly, even in this cohort, there were no correlation in the SNPs between SAFS, atopic asthma and healthy controls (64). These SNPs were specific to SAFS and there were major genetic differences even between closely related ABPA disease (64, 65). To fully appreciate the function of these variants in susceptibility to disease, it would be of great benefit to combine experimental models to delineate function of each variant in that gene. This was perfectly illustrated in patients with invasive Aspergillosis where variant rs35699176 in ZNF77 was shown to cause loss of integrity of the bronchial epithelium and allowed fungal growth (67). This variant was recapitulated *in vitro* by genetically-editing immortalized epithelial cells via CRISPR/cas9 which were subsequently infected with *A. fumigatus* conidia. Such studies in SAFS could lead to better understanding of gene function in disease susceptibility, especially in cases where human tissue is not easily accessible. It is worth noting that variants in the study by Knutsen et al. (63) were from 10 year old asthmatic children from multi-ethnic groups. This is relevant in Africa, where there are much larger genetic variations expected compared to Caucasian ancestry.

Several of the genetic polymorphisms associated with fungal allergic asthma are in the innate arm of the immune response to fungi, particularly pattern recognition receptors (PRRs) that recognize evolutionary conserved PAMPs. Although a full discussion is beyond this review, as we have recently reviewed this topic in great length (68), it is worth highlighting PRRs that are implicated in fungal induced asthma. Fungi is covered by cell wall which is mainly composed of outer mannan layer, a β-glucan layer and an inner chitin layer. β-glucan is a sugar found in most fungi and varies in content depending on the fungal species and stage of germination. Beta-glucan is recognized by Dectin-1 mainly by phagocytes and upon recognition initiates a cascade of events including phagocytosis, NF-κB activation, induction of pro-inflammatory genes including GM-CSF, TNF-α and activation of TH2 cytokine secreting cells. Studies in both human and mouse are contradictory regarding the role of Dectin-1 in the induction of TH2 cytokines (68). Several human studies support the idea that Dectin-1 engagement with its ligand favors TH2 environment either through instruction of naïve T cells to secrete TH2-type cytokines (69) or releasing of chemokines (CCL20) associated with TH2 cell recruitment (70). However, other studies suggest that Dectin-1 inhibits TH2 polarization through favoring a TH17 cell polarization or direct inhibition of IL-33 secretion (66, 69). In animal models, the role of Dectin-1 in allergic asthma is even more complicated, with several independent studies showing considerable inconsistences (54, 55, 71–76). Beta-glucan availability on the surface *Aspergillus versicolor* or *Cladosporium cladosporioides* live conidia determines the importance of Dectin-1 in TH2 airway immune responses (74). So, future studies should ascertain how each DECTIN-1 variant directly contributes to fungal induced asthma using both purified ligands and multiple fungal species with varying morphologies and growth stages as all these factors are likely to be important.

Toll-like receptors are another group of PRRs that have been shown to bind to fungi and induce pro-inflammatory cytokines (77–79). Several TLRs have been shown to recognize various PAMPs on the surface or released nucleic acid of fungi for example TLR2, TLR3, TLR4, TLR6, and TLR9 promote inflammation and clearance (80). TLRs can bind to fungal PAMPs individually or as heterodimeric clusters and have also been reported to cooperate with CLRs to induce immune responses (77, 81, 82). TLR3 SNP (rs10025405) rare G allele genotype results in susceptibility to SAFS. TLR3 is involved in direct recognition of *A. fumigatus* and upon recognition by epithelial cells activates a TRIF pathway leading to indoleamine 2,3-dioxygenase activation and cytokine production. Mice deficient of TLR-3 are susceptible to pulmonary *A. fumigatus* infection which may explain this susceptibility genotype (83). Currently, mechanisms of how TLR-3 or TLR3 SNP (rs100254050) can lead to susceptibility to severe asthma are unclear. TLR9 is an endosomal TLRs whose expression has been shown to be upregulated by Dectin-1 upon stimulation with *A. fumigatus* conidia or hyphae. TLR-9 regulates airway hyper-responsiveness and inflammation when sensitized and challenged with resting *A. fumigatus* conidia. Single nucleotide polymorphism in TLR-9 (rs352140) specifically the rare CC and CT genotypes are associated with SAFS (64). This suggests that TLR-9 plays an essential part in the modulating *A. fumigatus* induced allergic asthma. Mechanistically, it is likely that TLR-9 regulates allergic asthma induced by resisting *A. fumigatus* through regulating IL-5, IL-13, CCL11, and CCL21 and this process seems to be independent of Dectin-1 (84). During swollen *A. fumigatus* induced allergic asthma, TLR-9-deficient mice are more susceptible and show increased fungal growth. In this setting, TLR-9 completely regulates dectin-1 expression and by extension IL-17A expression, which explains increased fungal burden in the absence of TLR-9 (84).

Large birth cohort studies that primarily focus on the genetic-environmental interactions in the first year of life with follow-up of these infants up to at least 10 years of age can give greater insights into asthma development. Most risk genetic studies have ignored this crucial early phase where environmental exposure exerts most of its influence in the development of asthma. The importance of these studies was demonstrated in a 17q21 locus risk alleles ORMDL3 and GSDMB, where the same genotype associated with risk was influenced by environment to be protective (21). It would be of great interest to evaluate some of the fungal asthma associated risk alleles in birth cohort studies where environmental exposure at infancy if taken into account.

## How Is Allergy Tolerance Generated to Fungal Species?

Fungi is ubiquitous in our environment and it is estimated that in some seasons an individual can be exposed to as high as 50,000 spores per day, yet most individuals do not react and remain non-sensitized (49). Development of tolerance to fungal allergens is intricately regulated by a balance between regulatory T cells (Tregs) which suppress exaggerated inflammation and effector TH2 cells, which are essential in preventing invasive airway mycosis (85). Allergy is usually associated with loss of tolerance to highly allergen-specific TH2 cells and B cells producing IgE (86). In fungal induced TH2-type allergic airway responses, loss of tolerance to fungal allergens can be viewed as an active process aimed at limiting suppressive immune responses in favor of a robust TH2 airway inflammation capable of containing invasive and dissemination of infection to other organs such as the brain (27). How the immune system choses to react to some innocuous antigens or maintain tolerance to majority of inhaled airborne particles is poorly understood. This is particularly of great interest in fungal induced asthma where both active TH2 immunity and immunoregulation are required for initial host protection against invasive fungal species and limiting detrimental pathology. In fact most allergic individuals are extremely tolerant to majority of airborne allergens encountered, except for few that cause aberrant allergic disease (85, 87). Studies specifically focusing on T reg function in allergic neonates suggests an early dysfunction of these cells due to reduced expression CTLA4, TGF-β, resulting in failure in restraining a skewed thymic and peripheral TH2 cells (88). This is also supported by primary immune-deficiency studies in atopic neonates with immune dysregulation, polyendocrinopathy, enteropathy, X-linked (IPEX) syndrome where FOXP3 gene is impaired. These patients present with multiple organ autoimmunity and neonatal development of allergies due to unrestrained TH2 responses at mucosal surfaces (89). There are various mechanisms in which T reg cells can induce immune tolerance to allergens. These mechanisms do include active tolerance where both thymic or peripheral T regs actively suppress allergen specific naïve T cells, allergen ignorance and immunosuppression through secretion of IL-10 by Type 1 T regs (Tr1) (86, 89). The latter is not induced *de novo* and only seem to play a role in an already developed TH2 allergic disease, which makes it less attractive as therapeutic intervention in most allergic disease, but may well be beneficial in fungal induced asthma, which needs a balanced TH2 immunity and reduced host pathology (86). Two mechanisms seem to be relevant to fungal tolerance, namely direct suppression of allergen-specific TH2 cells by allergen specific T regs and allergen ignorance (85). The former is supported by studies using antigen reactive T cell-enrichment (ARTE) assays in sensitized individuals, where *A. fumigatus*-specific Treg cells directly compete with *A. fumigatus*-specific TH2 cells (85). Interestingly, this allergen suppression seems to be specific to particulate *A. fumigatus* allergens and rare in soluble allergens (85, 90). Allergen ignorance mechanisms of tolerance does suggest that most major fungal allergens are ignored by the immune system of healthy individuals. Studies using MHC multimer enrichment showed that allergen-specific T cells from healthy individuals were neither clonally expanding nor proliferating when exposed to antigen *in vitro* or during allergen season and resembled a naïve phenotype when compared to asthmatic individuals (91). Currently, it is unclear whether only certain fungal allergens are specific for inducing T regs or all allergens have a potential to induce both TH2 cells and T regs depending on the local cytokine milieu or antigen load. It is likely that pre-disposed individuals without disease possess a reasonable Treg compartment which keeps all airborne allergens at bay. However, during a low-grade fungal infection, this stable T reg compartment is highjacked leading to TH2 allergic immune response which keeps fungal load low and prevent dissemination (27, 85). This idea is supported by studies where chronic exposure to low grade *A. niger* is able to break tolerance induced by inert ovalbumin antigen in favor of a TH2 immune response (56). It is likely that there are numerous ways in which the human host has co-evolved with fungal airborne species and developed an intricately balanced tolerance vs. necessary inflammation to keep the pathogens out. It is likely that these tolerance mechanisms develop from birth and are nurtured throughout life, acting alone or in synergy with others to limit immunopathology. How tolerance induced by T reg cells toward common ubiquitous fungal airborne allergens is abrogated is of significant interest in the field of fungal induced asthma. Understanding mechanisms of tolerance to airborne fungi initiated by both neonatal and adult human thymic and peripheral Tregs would lead to not only new therapeutics that prevent this break of tolerance, but also interventions that limit immunopathology without compromising active fungal clearance. Appropriate mouse models that closely mimic the spectrum of fungal induced allergic asthma need to be developed in order to delineate mechanisms of tolerance.

## Fungal Asthma in Early-Life, How Can We Understand It?

Asthma is thought to be a disease that develops very early in life with almost a third of children under 5 years showing signs of wheezing. Lower respiratory infections contribute to poor lung function in infants who develop early onset of the disease. This early poor lung function is thought to persist until adulthood and is associated with premature mortality (20). Exposure to fungi in the first 2–3 months of life is generally associated with exacerbations of asthma in children (9, 22, 23, 30, 92). However, other studies in large birth cohorts that were followed from birth until age 13 have suggested that indoor fungal sensitization by certain dustborne yeasts were associated with protection (93). The mechanisms of protection to dustborne yeasts are unclear, but it is possible that because of hand-to-mouth feeding habits in infancy, these infants develop tolerance to such airborne yeast due to gut-specific immune responses that favor tolerance toward same ingested yeast (93). Other mechanisms of protection could be due to differences in species on dustborne vs. airborne fungal species which may also result in differential PAMPs exposure and subsequent stimulation of protective PRRs such as TLRs or C-type lectin receptors.

Lung is incompletely developed at birth and completes its post-natal development in the first 3 years of life (94, 95). This post-natal development is largely shaped by encountered external environment and means that the lung remains vulnerable to pathogen colonization during these first 3 years of life (96, 97). What is interesting about these early stages of life, is that pregnant mothers show a TH2 biased immune responses which is transferred to off springs who are also dominated by this skewed TH2 immunity (88). This inherited allergic status of infants from mothers is well-documented and is influenced by other factors including early exposure to microbes, cesarean delivery, breastfeeding, use of probiotics to name a few (96, 98). This TH2 skewed immune response reduces gradually during the first 2 years of life, however, allergic infants have been observed to retain this *utero* TH2 bias, which is thought to increase wheezing at this stage (99).

The data regarding immunological changes in the airways in early life is scarce and this is mainly due to invasive procedures required to get BAL samples or histopathology samples. This has also made it difficult to match clinical expression of asthma with changes in immune cells. The evidence which suggested both lung histopathological and physiological changes in children before age 3 who went on to develop asthma before pre-school gave some insights on early respiratory symptoms (100). Histopathological features of asthma have been observed after 3 years of age and include airway smooth muscle remodeling, variable reticular basement membrane and eosinophilic infiltration, a feature not seen in wheezing infants under 2 years old (100, 101). The lack of proper bronchoscopy control subjects in these studies makes it difficult to draw conclusions as it is unclear if this remodeling is due to the atopic disease or normal development of the lung. What also complicates these observations is that these infants are usually suspected of having severe respiratory symptoms and would be under corticosteroids, which may supress inflammatory cells such as eosinophils (96, 98).

To fully appreciate early determinants of asthma pathophysiology several studies have recapitulated these early events in animal models of allergic asthma (102–106). At steady state and during HDM exposure, innate lymphoid cells (ILC2s) producing IL-13 dominate the first 10 days of life in an ST2-dependent manner. This creates a TH2-biased immune phenotype, characterized by M2 macrophages that favor resolution of inflammation and protect the vulnerable developing lung (92, 102, 106). ILC2 have also been detected in sputum of children with severe form of asthma not responsive to corticosteroids, which suggests that these cells are essential in the early years in wheezing (107).

In humans, most studies in infants have mainly focussed on how certain bacterial taxa and viruses influence susceptibility to asthma in the first 3 years of life (96, 98). The information on fungal influence in the development of asthma at early stages is scarce and has only been looked at in the context of gut microbiome, where abundance of *Candida* and *Rhodotorula* species were associated with wheezing in 12 month old birth cohort (108). There are limited neonatal mouse studies focusing on how fungal species exacerbate allergic asthma. Those limited studies have solely focused on *A. alternata*, which is mainly associated with severe type of asthma, a disease not only restricted to children, but found in adults too. These studies have shown that *A. alternata* induces IL-33 dependent steroid resistant asthma (30, 105, 109, 110), which closely resemble that in children non-responsive to oral corticosteroids. This field is still in its infancy and more work is needed to combine asthma clinical data in pediatrics, fungal triggers in those environments and experimental neonatal immunology to decipher context specific disease mechanisms. Considering how little information is available in Africa on fungal sensitization and fungal species associated with different types of asthma, it is conceivable that disease mechanisms will differ, uncovering context specific drivers of allergic diseases. Below, we discuss some of the fungal species that have been associated with asthma.

## *Alternaria alternata* in Asthma

*Alternaria sp*. belongs to Ascomycota phylum which is the second most abundant fungal species. It is a cosmopolitan organism that is often isolated with outdoor environments, easily growing on dead vegetation or soil samples particularly during cultivation season of grainy plants (111). Alternaria can be found in rural and urban places particularly in warm climates and at higher CO_2_ levels, conditions that favor sporulation and high antigen content per spore (111). Spores have also been found in indoor environments in places such as basements or evaporative coolers (air-conditioning) where it is thought to be an important source of spores that induce exacerbations (48). Although sensitization to most fungal species is associated with asthma exacerbations, sensitization to *A. alternata* appears to increase the risk of potentially fatal asthma leading to higher hospital admissions under intensive care units (22, 45, 112). In a 25 year birth cohort prospective study done in the US, sensitization to *A. alternata* at age 6 was associated with increased hyperresponsiveness from ages 11 to 26 years, even in children who did not show signs of asthma (113). In another prospective study in Western Australia in 9-year-old children, *A. alternata* sensitization was found to strongly correlate with poor lung function when compared to sensitization to other allergens. Increases in AHR was associated with increase in *A. alternata* spores during dry season with spore counts reaching 300 spores/m^3^/day over 1 month (114). In the UK, Central and Eastern Europe, asthma exacerbations, high oral steroid usage and hospitalization have been linked with high *A. alternata* spore content and high allergen load (30, 31). Other retrospective studies have found as high as 20% *A. alternata* sensitization amongst mid-year school children from different locations in the US using more sensitive tests such as CAP system (44).

Most of what we know in terms of immune responses to *A. alternata* has mainly come from mouse studies, where TH2 cells have been demonstrated to play a superior role in the pathogenesis (30, 110, 115–117). In adult asthmatics or children directly sensitized or acutely exposed to *A. alternata*, immune responses have often not been done. In one pediatric study of children under 16 years, who were sensitized to *A. alternata* and had SAFS and unresponsive to oral steroids, they were found to have increased levels of IL-33 (30, 109). The mechanisms of steroid resistance induced by *A. alternata* were further evaluated in a neonatal mouse model where mice were chronically exposed to *A. alternata* and treated with steroids. IL-33 was found to be essential in *A. alternata* induced steroid resistant asthma by promoting TH2 cells and ILC2 that were not inhibited by steroids (30). Other studies have also shown a critical part of TH2 cells, ILC2, signaling molecule STAT6 and ST2 receptor in *A. alternata* induced allergic asthma (110, 115, 116, 118). Although these studies shed some light on immune mechanisms that lead to steroid resistance, it is currently unclear what factors within *A. alternata* organism leads to severe forms and fatal asthma. This is further complicated by lack of understanding on the function of major allergens, Alta 1 and 2. Serine proteases released by *A. alternata* have been shown to cleave IL-33, therefore leading to TH2 allergic airway responses (30, 110). More studies are needed from different geographic locations, socioeconomic status and age groups to elucidate the link between early sensitization to *A. alternata* exposure and development of steroid resistant asthma (44, 113). The function of innate immune responses and PRRs is less studied in both animal models and human challenge models, which can be explained by lack of studies looking at the cell wall properties of this environmentally important fungus.

## *Aspergillus* Species in Asthma

Up to 50% of asthmatics are also sensitized to fungi, presenting with increased IgE specific to fungi. The main culprit fungal species that are often found colonizing the lower airways of the asthmatic lung are *A. fumigatus* and other thermoresistant *Aspergillus* genera species (43). *A. fumigatus* is ubiquitous and is also found in airways of healthy people, where it does not cause any adverse effects and cleared. In *A. fumigatus* sensitized asthmatics, this fungus is associated with severe asthma which is characterized by frequent bronchiectasis, poor lung function and poor control of asthma. Sensitization to fungi, particularly *Aspergillus* with severe asthma symptoms has been classified as SAFS (43, 45, 119). Although SAFS is commonly used, however, this term is restricted to adult asthma and moderately used in children, partly because sensitization in children is often associated with immature immune system and difficulty in diagnoses. So, the term that has been adopted is fungal asthma (120).

*Aspergillus*-associated asthma is often masked or misdiagnosed with other *Aspergillus* associated diseases such as ABPA which in itself can be worsened by CPA (43, 119, 121, 122). Often, *Aspergillus* sensitized asthmatics do not meet the criteria for ABPA as their total IgE levels fall well-below threshold of (>410 IU/L or 1,000 ng/mL or >1,000 IU/L) and often present with normal levels of IgG (47, 121, 122). The other defining factors are lack of frequent pulmonary infections or bronchiectasis and fleeting shadows often seen in ABPA patients. CPA differs significantly from allergic asthma as it presents with morbidity associated features such as weight loss, productive cough, shortness of breath, pulmonary/pleural cavitation, localized fibrosis, and sometimes aspergilloma (47, 123). A few randomized clinical trials have shown a beneficial effect of antifungal drug (itraconazole) in more than 60% of patients with severe asthma sensitized to fungi, demonstrated by reduced IgE, exacerbations, and improved lung function and quality of life (124). Vericonazole, another antifungal drug have been shown to be effective against CPA (123), but had no effect in a randomized trial of moderate to severe asthmatics sensitized to *A. fumigatus* (125). Discontinuation of antifungal therapy is usually associated with rebound of asthma symptoms in both children and adults (124, 126), which highlights the difficulties in controlling environmental exposure to fungal allergens. Corticosteroids are commonly used to alleviate allergic asthma symptoms in SAFS, however they also pose a risk in developing invasive fungal dissemination (40). Immune responses to *A. fumigatus* associated sensitization are well-described in both animal models (28, 56) and human studies (127).

*A. fumigatus* lung infection is associated with TH2 cells, cytokines IL-4, IL-5, and IL-13, eosinophils and AHR. Severe form of the diseases is associated with TH17 cells, IL-17A and neutrophilic inflammation (128–130). However, some severe forms of the diseases are associated with hyper IgE and eosinophilia and resistance to corticosteroids (54). Recently, it was shown that there is a considerable cross-reactivity between *Candida albican*-specific TH17 cells residing in the gut and the vast majority of lung associated aero-allergens from *Aspergillus* spp. (127). These *C. albican*-specific T cells broadly promoted pathogenic TH17 cells against *A. fumigatus* in the lung and were associated with immunopathology. This gut/lung cross-talk has been observed in experimental mouse models where gut axis dysbiosis due to the use of antifungal drugs exacerbates allergic asthma (131, 132). What is characteristic of this lung responses is the proliferation of lung resident *A. fumigatus* and other aero-allergens as *C. albicans* disappear in the gut (131).

## *Cladosporium cladosporiodes* in Asthma

*Cladosporium* spp. are dematiaceous fungi and are found in a wide variety of habits, mainly foods. *Cladosporium* spp. are prevalent in indoor environments including moldy homes and are associated with severe forms of asthma particularly during sporulating season (133). *Cladosporium* spp. have been isolated in people's homes and air conditioners at work places and in some cases chronic exposure lead to hypersensitivity pneumonitis, hemorrhagic pneumonia, pulmonary fungal balls, left bronchial obstruction and lesions (32, 33, 134, 135). In some cases, *Cladosporium* spp. were associated with death, although it is unlikely that it was the sole cause of death, as some of the patients had underlying conditions such as diabetes milieus or cavitary lungs (32, 135). *Cladosporium cladosporiodes* is the major species detected in most atopic asthmatics followed by *Cladosporium herbarum*. Some studies have estimated that as high as 38% of atopic asthmatics are sensitized to *C. cladosporiodes*, which is in a similar range to other aero-allergens such as *Aspergillus* (136). This is based on immunoblot serum reactivity to major allergens such as serine protease (Cla c14) or peroxisomal protein (Clac c9) (Table 1). It is worth mentioning that there is a certain degree of overlap in amino acid sequence and IgE cross-reactivity between *C. cladosporiodes* and *Penicillium* spp., which can complicate diagnosis as both these species have a similar habitat and modes of human exposure (34). The mechanisms of how *Cladosporium* spp. or its individual components cause or exacerbate asthma are scarce and require further research. Some studies using secreted proteases suggest that proteases can activate allergic airway immune responses by cleaving key components of the lung epithelial barrier such as occludins resulting in a leaky barrier and more access for other allergens which leads to a co-ordinated local inflammatory cascade (137). It is also likely that *Cladosporium* spp. proteases act in a similar fashion to other fungal proteases by degrading specific endogenous substrates of which their products can bind directly to TLR-4 or to protease activated receptor 2 which then activate downstream signals through their cytoplasmic tails leading to recruitment of eosinophils, innate type 2 lymphoid cells and systemic IgE release (137–140). The importance of *C. cladosporiodes* proteases in the induction of allergic airway responses has been demonstrated in mouse models of allergic asthma (74, 75). In these models, chronic exposure of mice to live conidia induced a strong TH2 allergic airway disease characterized by eosinophilia, AHR, elevated levels of IgE and mucus production. However, when *C. cladosporiodes* conidia is inactivated by heat killing, it induced less AHR and allergic responses due to increased exposure of fungal β-glucan and Dectin-1 dependent neutrophilic and TH17 responses (74). These studies suggested that the factor that promotes TH2 allergic asthma in *C. cladosporiodes* where less β-glucan is exposed is likely to be proteases, although this was not formally investigated in this study.

## *Cryptococcus neoformans* in Asthma

*Cryptococcosis* is largely associated with immunocompromised patients and is the most common cause of HIV-related meningitis particularly in low CD4 count, killing over 150,000 people in Sub-Saharan Africa (141, 142). *Cryptococcus neoformans* and *Cryptococcus gattii*, are the causal agents. These are encapsulated basidiomycete found in the soil, tree bark and bird droppings. The spores or yeast form of fungus can easily be inhaled localizing in the alveoli space (37). M1 macrophages, TH1 and TH17 cells are essential in clearing infection (143). Although the immune response is sufficient to silence *Cryptococcus* to its dormancy, it is not sufficient to completely sterilize the lung (37, 143). Breakdown of immune defense leads to re-activation of this dormant stage and escape from lung, disseminating to other organs, particularly the brain (144). In immunocompetent individuals, *Cryptococcus* is thought to cause sub-clinical or asymptomatic pulmonary infections. This is often observed in urban areas that are densely populated and tend to have large pigeon colonies that live side-by-side with humans. Sub-clinical disease correlates with the presence of TH2 cells, M2 macrophages, and related cytokines and are detrimental in controlling dissemination and often associate with worse outcomes (38, 143, 145, 146). A few studies done in Northern America in Bronx area suggested that more than 50% of children over the age of 2 years were exposed to Cryptococcal antigens and their sera reacted to more than six antigens (147, 148). *Cryptococcus* exposure is associated with increased risk of developing asthma in children (147, 149). There is further evidence in mouse and rat animal models, where *Cryptococcus* infection exacerbates TH2 allergic airway inflammation (150). *Cryptococcus* virulent factors such as chitosan, proteases (Mpr1), laccases are the likely culprits in the development of TH2- type allergic asthma (Table 1), through favoring a defective immune arm (37). Encapsulation has been shown not to be essential in the initiation of TH2 type allergic asthma, despite its immune modulatory effects in IL-10 production and TH1 polarization (150, 151). Cryptococci cell wall is covered with chitin, which is known to have TH2-type stimulatory effects (37, 38). Human and mouse, both secrete Acidic Mammalian Chitinases (AMCase) known to digest huge chitin polymers and mediate TH2 airway immune responses (149, 152–154). Chitinases expression is increased in lung biopsies of adult asthmatics (152), although other studies have not shown comparable levels in BAL fluid (153). Children showing signs of exposure to Cryptococcal antigens, have elevated chitinases activity in the BAL fluid (149). A case study of a 10 year old boy also in the Bronx area who was sensitized to multiple fungal species including *C. neoformans* showed improvement in asthma symptoms such as reduced IgE and lung function when treated with itraconazole (126). Long term prospective studies are required to link early *Cryptococcus* exposure or infection to poor lung function and early acquisition of allergic asthma in children. What is currently known is that children are exposed to Cryptococcal antigen after the 2 years of life and make antibody responses to at least six antigens of similar nature to the ones observed in rat sera infected with *C. neoformans* (147, 150). The nature of these antigens is unclear and whether they can be used as diagnostic markers particularly for skin-prick tests or CAP to test for *Cryptococcus* sensitization. These studies should be strongly supported by experimental model studies to determine which Cryptococcal antigens are essential in establishing allergic asthma at an early age.

Fungal skin sensitization is common particularly in atopic dermatitis (AD) patients. AD patients are often pre-disposed to secondary allergic responses such as asthma. Atopic march is a term used to describe this progression from skin sensitization to allergic asthma (42). Here, we mention two fungal species that are mainly found colonizing the skin surface and often associated with AD and have been associated with allergic asthma. We briefly, discuss the mechanisms on how these skin resident fungi can cause allergic asthma.

## *Malassezia* in Atopic Dermatitis and Asthma

The skin serves as the first barrier to potential harm and is exposed to the largest number of pathogens due to its surface area, yet it contains the most diverse microbiome (155). Although large part of work has been focussed on bacterial species that colonize the skin such as *Staphylococcus epidermidis*, fungi accounts for almost a quarter of normal skin flora (156). *Malassezia* spp. colonize the skin of healthy and AD patients (156), but have also been found in sputum of asthmatic patients, which suggests their involvement in allergic asthma (35). *Malassezzia* spp. are dimorphic, lipophilic skin resident fungi that belongs to the phylum Basidiomycota, with at least 14 species identified so far in human and animal skin (36). The distribution of the species is geographically determined and largely driven by environment for example *Malassezia furfur* is more prevalent in Japan, whereas *Malassezia globota* and *Malassezia restricta* are found in Europe and US (36). *Malassezia* colonizes the skin as early as 0 and 1 days after birth and increases with time to almost 90% of skin being colonized by teenage years. However, there is considerable discrepancies in the species colonization between different studies (157, 158). A study by Lee et al. showed *M. restricta* to be common in teenagers and *M. globosa* predominant in adults over 50 years, whereas Gupta et al. found *M. globosa* to be more prevalent in younger subjects. *Malassezia* spp. is associated with at least three skin conditions including *Malassezia folliculitis*, Seborrheic dermatitis, *Pityriasis versicolor*, psoriasis and AD. The mechanisms on how *Malassezia* spp. contribute to any of these diseases are less defined and it is unclear if *Malassezia* spp. are the root causes of these conditions. In AD, *Malassezia* spp. is implicated as an aggravating factor, where it is thought that it releases allergens rather than being an infectious agent. The mechanisms of how *Malassezia* or its allergens promotes pathophysiology in AD patients is not well-defined, but is thought to be due to dysfunctional skin barrier, environmental factors, and other disturbances of normal skin flora, due to immunosuppression that favor mycelia growth over yeast form (36, 159). Once *Malassezia* spp. allergens enter the disrupted skin barrier, they are recognized by keratinocytes, Langerhan cells and other dermal dendritic cells that recognizes allergens and transport to local draining lymph nodes. This results in TH2 polarization and secretion of IL-4, IL-5, IL-13, and IgE which migrate back to the sight of inflammation further exacerbating AD. Other mechanisms of sensitization that have been suggested, are trafficking of *Malassezia* spp. allergens via extracellular vesicles (160). These extracellular vesicles (MalaEx) directly interact with keratinocytes and monocytes and are engulfed, becoming a major source of allergens, further exacerbating AD (Table 1) (160). *Malassezia* spp. sensitization (observed by *Malassezi*a specific IgE skin prick test) is less frequent in children compared to adults (161). *M. sympodialis* is the most frequently isolated species in AD skin lesions and patients on topical or systemic antifungal treatment show reduced severity of skin symptoms and IgE responses (161, 162). Both toll-like receptors (TLR-2 and TLR-4) and C-type lectin receptors, Mincle and Dectin-2 have been shown to bind specific ligands in *Malassezia* spp. and inducing specific pro-inflammatory cytokines in mouse macrophages (163, 164). However, these studies have not definitely shown whether these innate receptors promote *Malassezia*-specific IgE or promote IgE -independent skin inflammation that worsens AD.

## *Trichophyton interdigitale* in Asthma

*Trichophyton interdigitale* is a dermatophyte that can cause cutaneous infections when absorbed through the skin in millions of people worldwide. *Trichophyton* can also be inhaled, although it is unable to colonize the lung, it is associated with severe form of asthma (39). In some studies *T. interdigitale*-specific IgE responses correlate with severity of asthma and history of sensitization suggests childhood exposure (165, 166). *Trichophyton* and fungal skin infections are more common in immunocompromised people such as those with HIV infection, those receiving systemic corticosteroids and diabetes mellitus (39). In an African setting, there is significantly more people who are immunocompromised due to HIV and diabetes mellitus and develop skin infections, which may influence sensitization to *Trichophyton*. *Trichophyton* infections particularly scalp ringworm are predominant in children of African Caribbean or African American descent (167). In Africa, epidemiological data is scarce, however, these infections are likely to be high and probably involve other species of *Trichophyton* not reported in European, North American and other parts of the world. This early exposure to *Trichophyton* spp. certainly has profound impact on sensitization and development of allergies.

The immune responses to *Trichophyton spp*. is not well-defined and has somewhat remained an enigma with some anecdotes regarding its onset (39). It is believed that *Trichophyton* antigens are absorbed through barrier defected skin and these antigens are then taken up by local Langerhan antigen presenting dendritic cells. These antigen presenting cells then migrate to local draining lymph nodes and present antigens to T cells (39, 165). There are two main dominant immune responses to *Trichophyton* spp., an immediate hypersensitivity (IH) and delayed hypersensitivity (DHT) (39, 168). *Trichophyton*-specific IgE and IgG4 and mast cell activation are the main characteristics of IH and also involve urticaria and flare and these are generally associated with TH2 cell activation and cytokines such as IL-4, IL-5 and are localized (166). DHT response usually takes 48 h to come up and is correlated with presence of TH1 cells and IFN-γ production by these activated cells (168). What is intriguing about immune responses to *Trichophyton* is that some allergens can drive both IH and DHT and stimulate both TH2 and TH1 cell subsets (168). What also remains elusive is how *Trichophyton* skin sensitized individuals develop allergic airway asthma. Atopic march is a common phenomenon in allergic diseases and is likely at play where the skin colonizers become an important source of chronic exposure. However, this has not been investigated in *Trichophyton* sensitized individuals with asthma symptoms and animal models have not been investigated, where an allergen, APC and T cell can be tracked *in vivo*.

## Conclusions

Fungal species are ubiquitous, occupying at least 25% of Earth's biomass and are possibly found in every single niche of the human body cohabiting happily and forming part of the mucosal commensals. What is clear is that although the human body has learnt to tolerate fungi in most individuals, there are instances where this tolerance is dysregulated and cause various allergic pathologies. There is a lack of tools to detect fungal associated allergic sensitization and SPT for fungi are not routine. The use of artificial intelligence to predict IgE specific fungal allergens will likely have a bigger impact in hospital settings, particularly in prospective birth cohorts (169). In low- to middle-income countries, especially in Africa, these tools are currently beyond reach. Treatment options using both corticosteroids and antifungal drugs are promising but require further investigations. Antifungal drug access is also not widespread with some drugs either with no cheap generics, not available at all or only prescribed for life-threating infections such meningitis (170). Pharmacokinetics and drug-drug interactions between antifungal drugs and corticosteroids are not clear and require more randomized clinical trials in different population and age groups (120). Current FDA approved monoclonal therapy (omaluzimab or mepoluzimab) might be helpful in certain cases of severe hyper IgE, however, these are beyond reach for many African countries, so antifungal therapy remains the only attractive treatment method for fungal asthma (7, 120). It is important to note that only two countries in Africa have guidelines for asthma management in children, let alone fungal asthma (7, 171). Current SPT test in government hospitals do not include fungal allergens which makes it even harder to diagnose these asthma cases. There are limited studies focusing on the early development of fungal induced asthma and more work is needed to combine what is seen at clinics and experimental models. Long term birth cohort prospective studies are a valuable source of information regarding host genetics-environmental impact in the development of asthma (10, 172). In Africa, prospective birth cohorts to determine risk factors for asthma development are scarce (173, 174). More of these types of studies are needed to comprehensively evaluate impact of changing environment in the steady increase of asthma in Africa. Birth cohorts together with aerodynamic monitoring tools such as low-pressure impactor (ELPI) to specifically determine fungal allergen burden in indoor and outdoor environments will accelerate research in an African context and determine specific triggers of poor lung function and avoid a catastrophe (175).

## Author Contributions

SH and FB wrote the manuscript.

### Conflict of Interest

The authors declare that the research was conducted in the absence of any commercial or financial relationships that could be construed as a potential conflict of interest.

## References

[1] Anderson GP. Endotyping asthma: new insights into key pathogenic mechanisms in a complex, heterogeneous disease. Lancet. (2008) 372:1107–19. 10.1016/S0140-6736(08)61452-X 18805339

[2] Agustí A Noell G Brugada J Faner R. Lung function in early adulthood and health in later life: a transgenerational cohort analysis. Lancet Respir Med. (2017) 5:935–45. 10.1016/S2213-2600(17)30434-4 29150410

[3] Torgerson DG Ampleford EJ Chiu GY Gauderman WJ Gignoux CR Graves PE. Meta-analysis of genome-wide association studies of asthma in ethnically diverse North American populations. Nat Genet. (2011) 43:887–92. 10.1038/ng.888 21804549 PMC3445408

[4] Demenais F Margaritte-Jeannin P Barnes KC Cookson WOC Altmüller J Ang W. Multiancestry association study identifies new asthma risk loci that colocalize with immune-cell enhancer marks. Nat Genet. (2018) 50:42–50. 10.1038/s41588-017-0014-7 29273806 PMC5901974

[5] Ferreira MA Vonk JM Baurecht H Marenholz I Tian C Hoffman JD. Shared genetic origin of asthma, hay fever and eczema elucidates allergic disease biology. Nat Genet. (2017) 49:1752–7. 10.1038/ng.3985 29083406 PMC5989923

[6] Adeloye D Chan KY Rudan I Campbell H. An estimate of asthma prevalence in Africa: a systematic analysis. Croat Med J. (2013) 54:519–31. 10.3325/cmj.2013.54.519 24382846 PMC3893990

[7] Kwizera R Musaazi J Meya DB Worodria W Bwanga F Kajumbula H. Burden of fungal asthma in Africa: a systematic review and meta-analysis. PLoS ONE. (2019) 14:e0216568. 10.1371/journal.pone.0216568 31095641 PMC6521988

[8] Schuijs MJ Willart MA Vergote K Gras D Deswarte K Ege MJ. Farm dust and endotoxin protect against allergy through A20 induction in lung epithelial cells. Science. (2015) 349:1106–10. 10.1126/science.aac6623 26339029

[9] Stein MM Hrusch CL Gozdz J Igartua C Pivniouk V Murray SE. Innate immunity and asthma risk in amish and hutterite farm children. N Engl J Med. (2016) 375:411–21. 10.1056/NEJMoa1508749 27518660 PMC5137793

[10] Kirjavainen P V Karvonen AM Adams RI Täubel M Roponen M Tuoresmäki P Farm-like indoor microbiota in non-farm homes protects children from asthma development. Nat Med. (2019) 25:1089–95. 10.1038/s41591-019-0546-8 31209334 PMC7617062

[11] Wills-Karp M Santeliz J Karp CL. The germless theory of allergic disease: revisiting the hygiene hypothesis. Nat Rev Immunol. (2001) 1:69–75. 10.1038/35095579 11905816

[12] von Mutius E Vercelli D. Farm living: effects on childhood asthma and allergy. Nat Rev Immunol. (2010) 10:861–8. 10.1038/nri2871 21060319

[13] Ramsey CD Celedón JC. The hygiene hypothesis and asthma. Curr Opin Pulm Med. (2005) 11:14–20. 10.1097/01.mcp.0000145791.13714.ae 15591883

[14] Platts-Mills TAE Erwin E Heymann P Woodfolk J. Is the hygiene hypothesis still a viable explanation for the increased prevalence of asthma? Allergy Eur J Allergy Clin Immunol Suppl. (2005) 60:25–31. 10.1111/j.1398-9995.2005.00854.x 15842230

[15] Litonjua AA Milton DK Celedon JC Ryan L Weiss ST Gold DR. A longitudinal analysis of wheezing in young children: the independent effects of early life exposure to house dust endotoxin, allergens, and pets. J Allergy Clin Immunol. (2002) 110:736–42. 10.1067/mai.2002.128948 12417882

[16] Celedón JC Milton DK Ramsey CD Litonjua AA Ryan L Platts-Mills TA. Exposure to dust mite allergen and endotoxin in early life and asthma and atopy in childhood. J Allergy Clin Immunol. (2007) 120:144–9. 10.1016/j.jaci.2007.03.037 17507083 PMC3737770

[17] Ota MO van der Sande MA Walraven GE Jeffries D Nyan OA Marchant A. Absence of association between delayed type hypersensitivity to tuberculin and atopy in children in The Gambia. Clin Exp Allergy. (2003) 33:731–6. 10.1046/j.1365-2222.2003.01599.x 12801305

[18] Bager P Rostgaard K Nielsen NM Melbye M Westergaard T. Age at bacille Calmette–Guérin vaccination and risk of allergy and asthma. Clin Exp Allergy. (2003) 33:1512–7. 10.1046/j.1365-2222.2003.01796.x 14616862

[19] Uter W Stock C Pfahlberg A Guillén-Grima F Aguinaga-Ontoso I Brun-Sandiumenge C. Association between infections and signs and symptoms of ‘atopic' hypersensitivity – results of a cross-sectional survey among first-year university students in Germany and Spain. Allergy. (2003) 58:580–4. 10.1034/j.1398-9995.2003.00102.x 12823114

[20] Vasquez MM Zhou M Hu C Martinez FD Guerra S. Low lung function in young adult life is associated with early mortality. Am J Respir Crit Care Med. (2017) 195:1399–401. 10.1164/rccm.201608-1561LE 28504596 PMC5443902

[21] Loss GJ Depner M Hose AJ Genuneit J Karvonen AM Hyvärinen A. The early development of wheeze. environmental determinants and genetic susceptibility at 17q21. Am J Respir Crit Care Med. (2015) 193:889–97. 10.1164/rccm.201507-1493OC 26575599

[22] Black PN Udy AA Brodie SM. Sensitivity to fungal allergens is a risk factor for life-threatening asthma. Allergy. (2000) 55:501–4. 10.1034/j.1398-9995.2000.00293.x 10843433

[23] O'Driscoll BR Hopkinson LC Denning DW. Mold sensitization is common amongst patients with severe asthma requiring multiple hospital admissions. BMC Pulm Med. (2005) 5:4. 10.1186/1471-2466-5-4 15720706 PMC550663

[24] Weaver D Gago S Bromley M Bowyer P The human lung mycobiome in chronic respiratory disease: limitations of methods and our current understanding. Curr Fungal Infect Rep. (2019) 13:109–19. 10.1007/s12281-019-00347-5

[25] Tipton L Ghedin E Morris A. The lung mycobiome in the next-generation sequencing era. Virulence. (2017) 8:334–41. 10.1080/21505594.2016.1235671 27687858 PMC5436731

[26] Porter PC Ongeri V Luong A Kheradmand F Corry DB. Seeking common pathophysiology in asthma, atopy and sinusitis. Trends Immunol. (2011) 32:43–9. 10.1016/j.it.2010.11.007 21239229 PMC3042724

[27] Porter PC Lim DJ Maskatia ZK Mak G Tsai CL Citardi MJ. Airway surface mycosis in chronic TH2-associated airway disease. J Allergy Clin Immunol. (2014) 134:325–31.e9. 10.1016/j.jaci.2014.04.028 24928648 PMC4119856

[28] Porter P Susarla SC Polikepahad S Qian Y Hampton J Kiss A. Link between allergic asthma and airway mucosal infection suggested by proteinase-secreting household fungi. Mucosal Immunol. (2009) 2:504–17. 10.1038/mi.2009.102 19710638 PMC4115785

[29] Williams PB Barnes CS Portnoy JM Environmental Allergens Workgroup. Innate and adaptive immune response to fungal products and allergens. J Allergy Clin Immunol Pract. (2016) 4:386–95. 10.1016/j.jaip.2015.11.016 26755096

[30] Castanhinha S Sherburn R Walker S Gupta A Bossley CJ Buckley J. Pediatric severe asthma with fungal sensitization is mediated by steroid-resistant IL-33. J Allergy Clin Immunol. (2015) 136:312–22.e7. 10.1016/j.jaci.2015.01.016 25746970 PMC4534777

[31] Grewling Ł Nowak M Szymanska A Kostecki Ł Bogawski P. Temporal variability in the allergenicity of airborne *Alternaria* spores. Med Mycol. (2018) 57:403–11. 10.1093/mmy/myy069 30212862 PMC6441355

[32] Chiba S Okada S Suzuki Y Watanuki Z Mitsuishi Y Igusa R. *Cladosporium* species-related hypersensitivity pneumonitis in household environments. Intern Med. (2009) 48:363–7. 10.2169/internalmedicine.48.1811 19252363

[33] Grava S Lopes FAD Cavallazzi RS Grassi MFNN Svidzinski TIE. A rare case of hemorrhagic pneumonia due to *Cladosporium cladosporioides*. J Bras Pneumol. (2016) 42:392–394. 10.1590/S1806-37562016000000079 27812642 PMC5094879

[34] Chou H Tam MF Chiang C-H Chou C-T Tai H-Y Shen H-D. Transaldolases are novel and immunoglobulin E cross-reacting fungal allergens. Clin Exp Allergy. (2011) 41:739–49. 10.1111/j.1365-2222.2011.03698.x 21488999

[35] van Woerden HC Gregory C Brown R Marchesi JR Hoogendoorn B Matthews IP. Differences in fungi present in induced sputum samples from asthma patients and non-atopic controls: a community based case control study. BMC Infect Dis. (2013) 13:69. 10.1186/1471-2334-13-69 23384395 PMC3570489

[36] Prohic A Jovovic Sadikovic T Krupalija-Fazlic M Kuskunovic-Vlahovljak S. Malassezia species in healthy skin and in dermatological conditions. Int J Dermatol. (2016) 55:494–504. 10.1111/ijd.13116 26710919

[37] Wozniak KL Olszewski MA Wormley FL. Molecules at the interface of *Cryptococcus* and the host that determine disease susceptibility. Fungal Genet Biol. (2015) 78:87–92. 10.1016/j.fgb.2014.10.013 25445308

[38] Wiesner DL Specht CA Lee CK Smith KD Mukaremera L Lee ST. Chitin recognition via chitotriosidase promotes pathologic type-2 helper T cell responses to cryptococcal infection. PLoS Pathog. (2015) 11:1–28. 10.1371/journal.ppat.1004701 25764512 PMC4357429

[39] Woodfolk JA Allergy and dermatophytes. Clin Microbiol Rev. (2005) 2005:30–43. 10.1128/CMR.18.1.30-43.2005 PMC544172 15653817

[40] Fraczek MG Chishimba L Niven RM Bromley M Simpson A Smyth L. Corticosteroid treatment is associated with increased filamentous fungal burden in allergic fungal disease. J Allergy Clin Immunol. (2018) 142:407–14. 10.1016/j.jaci.2017.09.039 29122659

[41] Luong A Davis LS Marple BF. Peripheral blood mononuclear cells from allergic fungal rhinosinusitis adults express a Th_2_ cytokine response to fungal antigens. Am J Rhinol Allergy. (2009) 23:281–7. 10.2500/ajra.2009.23.3311 19490802

[42] Spergel JM. From atopic dermatitis to asthma: the atopic march. Ann Allergy Asthma Immunol. (2010) 105:99–106. 10.1016/j.anai.2009.10.002 20674819

[43] Knutsen AP Bush RK Demain JG Denning DW Dixit A Fairs A. Fungi and allergic lower respiratory tract diseases. J Allergy Clin Immunol. (2012) 129:280–91. 10.1016/j.jaci.2011.12.970 22284927

[44] Perzanowski MS Sporik R Squillace SP Gelber LE Call R Carter M. Association of sensitization to Alternaria allergens with asthma among school-age children. J Allergy Clin Immunol. (1998) 101:626–32. 10.1016/S0091-6749(98)70170-8 9600499

[45] Denning DW O'Driscoll BR Hogaboam CM Bowyer P Niven RM. The link between fungi and severe asthma: a summary of the evidence. Eur Respir J. (2006) 27:615–26. 10.1183/09031936.06.00074705 16507864

[46] Green BJ Tovey ER Sercombe JK Blachere FM Beezhold DH Schmechel D. Airborne fungal fragments and allergenicity. Med Mycol. (2006) 44(Suppl. 1):245–55. 10.1080/13693780600776308 17050446

[47] Denning DW Pashley C Hartl D Wardlaw A Godet C Del Giacco S. Fungal allergy in asthma–state of the art and research needs. Clin Transl Allergy. (2014) 4:14. 10.1186/2045-7022-4-14 24735832 PMC4005466

[48] Pringle A. Asthma and the diversity of fungal spores in air. PLoS Pathog. (2013) 9:1–4. 10.1371/journal.ppat.1003371 23762024 PMC3675135

[49] Cho SH Seo SC Schmechel D Grinshpun SA Reponen T Aerodynamic characteristics and respiratory deposition of fungal fragments. Atmos Environ. (2005) 39:5454–65. 10.1016/j.atmosenv.2005.05.042

[50] Fröhlich-Nowoisky J Pickersgill DA Després VR Pöschl U. High diversity of fungi in air particulate matter. Proc Natl Acad Sci USA. (2009) 106:12814–9. 10.1073/pnas.0811003106 19617562 PMC2722276

[51] Karim M Sheikh H Alam M Sheikh Y. Disseminated bipolaris infection in an asthmatic patient: case report. Clin Infect Dis. (1993) 17:248–53. 10.1093/clinids/17.2.248 8399876

[52] Ribeiro NFF Cousin GCS Wilson GE Butterworth DM Woodwards RTM. Lethal invasive mucormycosis: case report and recommendations for treatment. Int J Oral Maxillofac Surg. (2001) 30:156–9. 10.1054/ijom.2000.0010 11405452

[53] Chishimba L Niven RM Cooley J Denning DW. Voriconazole and posaconazole improve asthma severity in allergic bronchopulmonary aspergillosis and severe asthma with fungal sensitization. J Asthma. (2012) 49:423–33. 10.3109/02770903.2012.662568 22380765

[54] Zhang Z Biagini Myers JM Brandt EB Ryan PH Lindsey M Mintz-Cole RA. β-Glucan exacerbates allergic asthma independent of fungal sensitization and promotes steroid-resistant TH2/TH17 responses. J Allergy Clin Immunol. (2017) 139:54–65.e8. 10.1016/j.jaci.2016.02.031 27221135 PMC5073040

[55] Hadebe S Kirstein F Fierens K Redelinghuys P Murray GI Williams DL. β-Glucan exacerbates allergic airway responses to house dust mite allergen. Respir Res. (2016) 17:35. 10.1186/s12931-016-0352-5 27039089 PMC4818888

[56] Porter PC Roberts L Fields A Knight M Qian Y Delclos GL. Necessary and sufficient role for T helper cells to prevent fungal dissemination in allergic lung disease. Infect Immun. (2011) 79:4459–71. 10.1128/IAI.05209-11 21875960 PMC3257943

[57] Kauffman HF van der Heide S. Exposure, sensitization, and mechanisms of fungus-induced asthma. Curr Allergy Asthma Rep. (2003) 3:430–7. 10.1007/s11882-003-0080-z 12906782

[58] Barnes C. Fungi and atopy. Clin Rev Allergy Immunol. (2019) 1–10. 10.1007/s12016-019-08750-z 31321665

[59] Massoud AH Charbonnier L Lopez D Pellegrini M Phipatanakul W Chatila TA An asthma-associated IL4R variant exacerbates airway inflammation by promoting conversion of regulatory T cells to T H 17-like cells. Nat Med. (2016) 22:1–13. 10.1038/nm.4147 27479084 PMC5014738

[60] Hershey GKK Friedrich MF Esswein LA Thomas ML Chatila TA. The association of atopy with a gain-of-function mutation in the α subunit of the interleukin-4 receptor. N Engl J Med. (1997) 337:1720–5. 10.1056/NEJM199712113372403 9392697

[61] Ober C Yao TC. The genetics of asthma and allergic disease: a 21st century perspective. Immunol Rev. (2011) 242:10–30. 10.1111/j.1600-065X.2011.01029.x 21682736 PMC3151648

[62] Ober C Leavitt SA Tsalenko A Howard TD Hoki DM Daniel R. Variation in the interleukin 4–receptor α gene confers susceptibility to asthma and atopy in ethnically diverse populations. Am J Hum Genet. (2000) 66:517–26. 10.1086/302781 10677312 PMC1288105

[63] Knutsen AP Vijay HM Kariuki B Santiago LA Graff R Wofford JD. Association of IL-4RA single nucleotide polymorphisms, HLA-DR and HLA-DQ in children with Alternaria-sensitive moderate-severe asthma. Clin Mol Allergy. (2010) 8:5. 10.1186/1476-7961-8-5 20298583 PMC2846861

[64] Overton NL Simpson A Bowyer P Denning DW. Genetic susceptibility to severe asthma with fungal sensitization. Int J Immunogenet. (2017) 44:93–106. 10.1111/iji.12312 28371335

[65] Overton NLD Denning DW Bowyer P Simpson A. Genetic susceptibility to allergic bronchopulmonary aspergillosis in asthma: a genetic association study. Allergy Asthma Clin Immunol. (2016) 12:47. 10.1186/s13223-016-0152-y 27708669 PMC5037889

[66] Gour N Lajoie S Smole U White M Hu D Goddard P. Dysregulated invertebrate tropomyosin–dectin-1 interaction confers susceptibility to allergic diseases. Sci Immunol. (2018) 3:eaam9841. 10.1126/sciimmunol.aam9841 29475849 PMC5956913

[67] Gago S Overton NLD Ben-Ghazzi N Novak-Frazer L Read ND Denning DW. Lung colonization by Aspergillus fumigatus is controlled by ZNF77. Nat Commun. (2018) 9:3835. 10.1038/s41467-018-06148-7 30237437 PMC6147781

[68] Hadebe S Brombacher F Brown GD. C-type lectin receptors in asthma. Front Immunol. (2018) 9:733. 10.3389/fimmu.2018.00733 29696023 PMC5904212

[69] Joo H Upchurch K Zhang W Ni L Li D Xue Y. Opposing roles of dectin-1 expressed on human plasmacytoid dendritic cells and myeloid dendritic cells in Th_2_ polarization. J Immunol. (2015) 195:1723–31. 10.4049/jimmunol.1402276 26123355 PMC4530104

[70] Nathan AT Peterson EA Chakir J Wills-Karp M. Innate immune responses of airway epithelium to house dust mite are mediated through β-glucan-dependent pathways. J Allergy Clin Immunol. (2009) 123:612–8. 10.1016/j.jaci.2008.12.006 19178937 PMC2761684

[71] Werner JL Gessner MA Lilly LM Nelson MP Metz AE Horn D. Neutrophils produce interleukin 17A (IL-17A) in a Dectin-1- and IL-23-dependent manner during invasive fungal infection. Infect Immun. (2011) 79:3966–77. 10.1128/IAI.05493-11 21807912 PMC3187263

[72] Lilly LM Gessner MA Dunaway CW Metz AE Schwiebert L Weaver CT. The β-glucan receptor dectin-1 promotes lung immunopathology during fungal allergy via IL-22. J Immunol. (2012) 189:3653–60. 10.4049/jimmunol.1201797 22933634 PMC3448838

[73] Ryu JH Yoo JY Kim MJ Hwang SG Ahn KC Ryu JC. Distinct TLR-mediated pathways regulate house dust mite-induced allergic disease in the upper and lower airways. J Allergy Clin Immunol. (2013) 131:549–61. 10.1016/j.jaci.2012.07.050 23036747

[74] Mintz-Cole RA Brandt EB Bass SA Gibson AM Reponen T Khurana Hershey GK. Surface availability of beta-glucans is critical determinant of host immune response to *Cladosporium cladosporioides*. J Allergy Clin Immunol. (2013) 132:159–69. 10.1016/j.jaci.2013.01.003 23403046 PMC6145803

[75] Mintz-Cole RA Gibson AM Bass SA Budelsky AL Reponen T Hershey GKK Dectin-1 and IL-17A suppress murine asthma induced by Aspergillus versicolor but not *Cladosporium cladosporioides* due to differences in β-glucan surface exposure. J Immunol. (2012) 189:3609–17. 10.4049/jimmunol.1200589 22962686 PMC3470885

[76] Ito T Hirose K Norimoto A Tamachi T Yokota M Saku A. Dectin-1 plays an important role in house dust mite-induced allergic airway inflammation through the activation of CD11b+ dendritic cells. J Immunol. (2017) 198:61–70. 10.4049/jimmunol.1502393 27852745

[77] Netea MG Gow NA Munro CA Bates S Collins C Ferwerda G. Immune sensing of Candida albicans requires cooperative recognition of mannans and glucans by lectin and Toll-like receptors. J Clin Invest. (2006) 116:1642–50. 10.1172/JCI27114 16710478 PMC1462942

[78] Ferwerda G Meyer-Wentrup F Kullberg BJ Netea MG Adema GJ. Dectin-1 synergizes with TLR2 and TLR4 for cytokine production in human primary monocytes and macrophages. Cell Microbiol. (2008) 10:2058–66. 10.1111/j.1462-5822.2008.01188.x 18549457

[79] Hohl TM Van Epps HL Rivera A Morgan LA Chen PL Feldmesser M. *Aspergillus fumigatus* triggers inflammatory responses by stage-specific β-glucan display. PLoS Pathog. (2005) 1:e30. 10.1371/journal.ppat.0010030 16304610 PMC1287910

[80] Mambula SS Sau K Henneke P Golenbock DT Levitz SM. Toll-like receptor (TLR) signaling in response to *Aspergillus fumigatus*. J Biol Chem. (2002) 277:39320–6. 10.1074/jbc.M201683200 12171914

[81] Sousa Mda G Reid DM Schweighoffer E Tybulewicz V Ruland J Langhorne J. Restoration of pattern recognition receptor costimulation to treat chromoblastomycosis, a chronic fungal infection of the skin. Cell Host Microbe. (2011) 9:436–43. 10.1016/j.chom.2011.04.005 21575914 PMC3098964

[82] Dennehy KM Ferwerda G Faro-Trindade I Pyz E Willment JA Taylor PR. Syk kinase is required for collaborative cytokine production induced through Dectin-1 and Toll-like receptors. Eur J Immunol. (2008) 38:500–6. 10.1002/eji.200737741 18200499 PMC2430329

[83] Carvalho A De Luca A Bozza S Cunha C D'Angelo C Moretti S. TLR3 essentially promotes protective class I–restricted memory CD8+ T-cell responses to Aspergillus fumigatus in hematopoietic transplanted patients. Blood. (2012) 119:967–77. 10.1182/blood-2011-06-362582 22147891

[84] Ramaprakash H Ito T Standiford TJ Kunkel SL Hogaboam CM. Toll-like receptor 9 modulates immune responses to *Aspergillus fumigatus* conidia in immunodeficient and allergic mice. Infect Immun. (2009) 77:108–19. 10.1128/IAI.00998-08 18936185 PMC2612288

[85] Bacher P Heinrich F Stervbo U Nienen M Vahldieck M Iwert C. Regulatory T cell specificity directs tolerance versus allergy against *Aeroantigens* in humans. Cell. (2016) 167:1067–78.e16. 10.1016/j.cell.2016.09.050 27773482

[86] Bacher P Scheffold A. The effect of regulatory T cells on tolerance to airborne allergens and allergen immunotherapy. J Allergy Clin Immunol. (2018) 142:1697–709. 10.1016/j.jaci.2018.10.016 30527063

[87] Palm NW Rosenstein RK Medzhitov R. Allergic host defences. Nature. (2012) 484:465–72. 10.1038/nature11047 22538607 PMC3596087

[88] Tulic MK Andrews D Crook ML Charles A Tourigny MR Moqbel R. Changes in thymic regulatory T-cell maturation from birth to puberty: differences in atopic children. J Allergy Clin Immunol. (2012) 129:199–206.e4. 10.1016/j.jaci.2011.10.016 22104606

[89] Sakaguchi S Yamaguchi T Nomura T Ono M. Regulatory T cells and immune tolerance. Cell. (2008) 133:775–87. 10.1016/j.cell.2008.05.009 18510923

[90] Bacher P Kniemeyer O Schönbrunn A Sawitzki B Assenmacher M Rietschel E. Antigen-specific expansion of human regulatory T cells as a major tolerance mechanism against mucosal fungi. Mucosal Immunol. (2013) 7:916. 10.1038/mi.2013.107 24301658

[91] Wambre E Bajzik V DeLong JH O'Brien K Nguyen QA Speake C. A phenotypically and functionally distinct human TH_2_ cell subpopulation is associated with allergic disorders. Sci Transl Med. (2017) 9:eaam9171. 10.1126/scitranslmed.aam9171 28768806 PMC5987220

[92] Bachus H Kaur K Papillion AM Marquez-Lago TT Yu Z Ballesteros-Tato A. Impaired tumor-necrosis-factor-α-driven dendritic cell activation limits lipopolysaccharide-induced protection from allergic inflammation in infants. Immunity. (2019) 50:225–40.e4. 10.1016/j.immuni.2018.11.012 30635238 PMC6335154

[93] Behbod B Sordillo JE Hoffman EB Datta S Webb TE Kwan DL. Asthma and allergy development: contrasting influences of yeasts and other fungal exposures. Clin Exp Allergy. (2015) 45:154–63. 10.1111/cea.12401 25200568 PMC4733530

[94] Metzger RJ Klein OD Martin GR Krasnow MA. The branching programme of mouse lung development. Nature. (2008) 453:745–50. 10.1038/nature07005 18463632 PMC2892995

[95] Treutlein B Brownfield DG Wu AR Neff NF Mantalas GL Espinoza FH. Reconstructing lineage hierarchies of the distal lung epithelium using single-cell RNA-seq. Nature. (2014) 509:371–5. 10.1038/nature13173 24739965 PMC4145853

[96] Lloyd CM Saglani S. Development of allergic immunity in early life. Immunol Rev. (2017) 278:101–15. 10.1111/imr.12562 28658545

[97] Guerra S Martinez FD. Epidemiology of the origins of airflow limitation in asthma. Proc Am Thorac Soc. (2009) 6:707–11. 10.1513/pats.200908-085DP 20008881 PMC2797072

[98] Jackson DJ Gern JE Lemanske RF. Lessons learned from birth cohort studies conducted in diverse environments. J Allergy Clin Immunol. (2017) 139:379–86. 10.1016/j.jaci.2016.12.941 28183432 PMC5508737

[99] Prescott SL Macaubas C Smallacombe T Holt BJ Sly PD Loh R. Reciprocal age-related patterns of allergen-specific T-cell immunity in normal vs. atopic infants. Clin Exp Allergy. (1998) 28(suppl. 5):39–44. 10.1046/j.1365-2222.1998.028s5039.x 9988446

[100] Saglani S Malmström K Pelkonen AS Malmberg LP Lindahl H Kajosaari M. Airway remodeling and inflammation in symptomatic infants with reversible airflow obstruction. Am J Respir Crit Care Med. (2005) 171:722–7. 10.1164/rccm.200410-1404OC 15657459

[101] Lezmi G Gosset P Deschildre A Abou-Taam R Mahut B Beydon N. Airway remodeling in preschool children with severe recurrent wheeze. Am J Respir Crit Care Med. (2015) 192:164–71. 10.1164/rccm.201411-1958OC 25961111

[102] de Kleer IM Kool M de Bruijn MJ Willart M van Moorleghem J Schuijs MJ. Perinatal activation of the interleukin-33 pathway promotes type 2 immunity in the developing lung. Immunity. (2016) 45:1285–98. 10.1016/j.immuni.2016.10.031 27939673

[103] Trompette A Gollwitzer ES Yadava K Sichelstiel AK Sprenger N Ngom-Bru C. Gut microbiota metabolism of dietary fiber influences allergic airway disease and hematopoiesis. Nat Med. (2014) 20:159–66. 10.1038/nm.3444 24390308

[104] Gollwitzer ES Saglani S Trompette A Yadava K Sherburn R McCoy KD. Lung microbiota promotes tolerance to allergens in neonates via PD-L1. (2014) 20:3568. 10.1038/nm.3568 24813249

[105] Saglani S Gregory LG Manghera AK Branchett WJ Uwadiae F Entwistle LJ. Inception of early-life allergen–induced airway hyperresponsiveness is reliant on IL-13 + CD4 + T cells. Sci Immunol. (2018) 3:eaan4128. 10.1126/sciimmunol.aan4128 30194239 PMC7613599

[106] Saluzzo S Gorki AD Rana BMJ Martins R Scanlon S Starkl P. First-breath-induced type 2 pathways shape the lung immune environment. Cell Rep. (2017) 18:1893–905. 10.1016/j.celrep.2017.01.071 28228256 PMC5329122

[107] Nagakumar P Denney L Fleming L Bush A Lloyd CM Saglani S. Type 2 innate lymphoid cells in induced sputum from children with severe asthma. J Allergy Clin Immunol. (2016) 137:624–6.e6. 10.1016/j.jaci.2015.06.038 26277593

[108] Fujimura KE Sitarik AR Havstad S Lin DL Levan S Fadrosh D. Neonatal gut microbiota associates with childhood multisensitized atopy and T cell differentiation. Nat Med. (2016) 22:1187. 10.1038/nm.4176 27618652 PMC5053876

[109] Saglani S Lui S Ullmann N Campbell GA Sherburn RT Mathie SA. IL-33 promotes airway remodeling in pediatric patients with severe steroid-resistant asthma. J Allergy Clin Immunol. (2013) 132:676–85.e13. 10.1016/j.jaci.2013.04.012 23759184 PMC4218948

[110] Snelgrove RJ Gregory LG Peiró T Akthar S Campbell GA Walker SA. *Alternaria*-derived serine protease activity drives IL-33-mediated asthma exacerbations. J Allergy Clin Immunol. (2014) 134:583–92.e6. 10.1016/j.jaci.2014.02.002 24636086 PMC4152000

[111] Wolf J O'Neill RN Rogers AC Muilenberg LM Ziska HL. Elevated atmospheric carbon dioxide concentrations amplify alternaria alternata sporulation and total antigen production. Environ Health Perspect. (2010) 118:1223–8. 10.1289/ehp.0901867 20462828 PMC2944081

[112] Bush RK Prochnau JJ. Alternaria-induced asthma. J Allergy Clin Immunol. (2004) 113:227–34. 10.1016/j.jaci.2003.11.023 14767434

[113] Hiranrattana A Stern DA Guerra S Halonen M Wright AL Daines M. Alternaria sensitisation at age 6 years is associated with subsequent airway hyper-responsiveness in non-asthmatics. Thorax. (2018) 73:1170–3. 10.1136/thoraxjnl-2017-210325 29563159 PMC6410363

[114] Downs SH Mitakakis TZ Marks GB Car NG Belousova EG Leüppi JD. Clinical importance of alternaria exposure in children. Am J Respir Crit Care Med. (2001) 164:455–9. 10.1164/ajrccm.164.3.2008042 11500349

[115] Doherty TA Khorram N Sugimoto K Sheppard D Rosenthal P Cho JY. *Alternaria* induces STAT6-dependent acute airway eosinophilia and epithelial FIZZ1 expression that promotes airway fibrosis and epithelial thickness. J Immunol. (2012) 188:2622–9. 10.4049/jimmunol.1101632 22327070 PMC3294141

[116] Doherty TA Khorram N Lund S Mehta AK Croft M Broide DH. Lung type 2 innate lymphoid cells express cysteinyl leukotriene receptor 1, which regulates TH_2_ cytokine production. J Allergy Clin Immunol. (2013) 132:205–13. 10.1016/j.jaci.2013.03.048 23688412 PMC3704056

[117] Löser S Gregory LG Zhang Y Schaefer K Walker SA Buckley J. Pulmonary ORMDL3 is critical for induction of *Alternaria*-induced allergic airways disease. J Allergy Clin Immunol. (2017) 139:1496–507.e3. 10.1016/j.jaci.2016.07.033 27623174 PMC5415707

[118] Valladao AC Frevert CW Koch LK Campbell DJ Ziegler SF. STAT6 regulates the development of eosinophilic versus neutrophilic asthma in response to *Alternaria alternata*. J Immunol. (2016) 197:4541–51. 10.4049/jimmunol.1600007 27815425 PMC5136320

[119] Denning DW Pleuvry A Cole DC. Global burden of allergic bronchopulmonary aspergillosis with asthma and its complication chronic pulmonary aspergillosis in adults. Med Mycol. (2013) 51:361–70. 10.3109/13693786.2012.738312 23210682

[120] Moss RB. Treatment options in severe fungal asthma and allergic bronchopulmonary aspergillosis. Eur Respir J. (2014) 43:1487–500. 10.1183/09031936.00139513 24311776

[121] Agarwal R Nath A Aggarwal AN Gupta D Chakrabarti A. Aspergillus hypersensitivity and allergic bronchopulmonary aspergillosis in patients with acute severe asthma in a respiratory intensive care unit in North India. Mycoses. (2010) 53:138–43. 10.1111/j.1439-0507.2008.01680.x 19207831

[122] Agarwal R Gupta D. Severe asthma and fungi: current evidence. Med Mycol. (2011) 49(Suppl 1):150–7. 10.3109/13693786.2010.504752 20662637

[123] Camuset J Nunes H Dombret MC Bergeron A Henno P Philippe B. Treatment of chronic pulmonary aspergillosis by voriconazole in nonimmunocompromised patients. Chest. (2007) 131:1435–41. 10.1378/chest.06-2441 17400661

[124] Denning DW O'Driscoll BR Powell G Chew F Atherton GT Vyas A. Randomized controlled trial of oral antifungal treatment for severe asthma with fungal sensitization: the Fungal Asthma Sensitization Trial (FAST) study. Am J Respir Crit Care Med. (2009) 179:11–8. 10.1164/rccm.200805-737OC 18948425

[125] Agbetile J Bourne M Fairs A Hargadon B Desai D Broad C. Effectiveness of voriconazole in the treatment of *Aspergillus fumigatus*–associated asthma (EVITA3 study). J Allergy Clin Immunol. (2014) 134:33–9. 10.1016/j.jaci.2013.09.050 24290286

[126] Vicencio AG Muzumdar H Tsirilakis K Kessel A Nandalike K Goldman DL. Severe asthma with fungal sensitization in a child: response to itraconazole therapy. Pediatrics. (2010) 125:1255–8. 10.1542/peds.2009-2443 20385639

[127] Bacher P Hohnstein T Beerbaum E Röcker M Blango MG Kaufmann S. Human anti-fungal Th17 immunity and pathology rely on cross-reactivity against *Candida albicans*. Cell. (2019) 176:1340–55.e15. 10.1016/j.cell.2019.01.041 30799037

[128] Chen F Liu Z Wu W Rozo C Bowdridge S Millman A An essential role for the Th2-type response in limiting tissue damage during helmit infection. Natl Inst Heal. (2012) 18:260–6. 10.1038/nm.2628 PMC3274634 22245779

[129] Fei M Bhatia S Oriss TB Yarlagadda M Khare A Akira S. TNF-alpha from inflammatory dendritic cells (DCs) regulates lung IL-17A/IL-5 levels and neutrophilia versus eosinophilia during persistent fungal infection. Proc Natl Acad Sci USA. (2011) 108:5360–5. 10.1073/pnas.1015476108 21402950 PMC3069210

[130] Hadebe S Kirstein F Fierens K Chen K Drummond RA Vautier S Microbial ligand costimulation drives neutrophilic steroid-refractory asthma. PLoS ONE. (2015) 10:e0137945 10.1371/journal.pone.0137945 26261989 PMC4532492

[131] Wheeler ML Limon JJ Bar AS Leal CA Gargus M Tang J. Immunological consequences of intestinal fungal dysbiosis. Cell Host Microbe. (2016) 19:865–73. 10.1016/j.chom.2016.05.003 27237365 PMC4900921

[132] Li X Leonardi I Semon A Doron I Gao IH Putzel GG. Response to fungal dysbiosis by gut-resident CX3CR1+ mononuclear phagocytes aggravates allergic airway disease. Cell Host Microbe. (2018) 24:847–56.e4. 10.1016/j.chom.2018.11.003 30503509 PMC6292739

[133] Chowdhary A Agarwal K Kathuria S Gaur SN Randhawa HS Meis JF. Allergic bronchopulmonary mycosis due to fungi other than *Aspergillus*: a global overview. Crit Rev Microbiol. (2014) 40:30–48. 10.3109/1040841X.2012.754401 23383677

[134] Feldman GJ Mucoid lesion obstructing left main bronchus associated with isolation of cladosporium fungal species. J Bronchology Interv Pulmonol. (1999) 6:9 10.1097/00128594-199907000-00009

[135] Kwon-Chung KJ Schwartz IS Rybak BJ. A pulmonary fungus ball produced by *Cladosporium cladosporioides*. Am J Clin Pathol. (1975) 64:564–8. 10.1093/ajcp/64.4.564 1239189

[136] Chou H Tam MF Lee LH Chiang CH Tai HY Panzani RC. Vacuolar serine protease is a major allergen of *Cladosporium cladosporioides*. Int Arch Allergy Immunol. (2008) 146:277–86. 10.1159/000121462 18362473

[137] Li E Knight JM Wu Y Chapter four–airway mycosis in allergic airway disease. Adv Immunol. (2019) 142:85–140. 10.1016/bs.ai.2019.05.002 31296304

[138] Millien VO Lu W Shaw J Yuan X Mak G Roberts L. Cleavage of fibrinogen by proteinases elicits allergic responses through Toll-like receptor 4. Science. (2013) 341:792–6. 10.1126/science.1240342 23950537 PMC3898200

[139] Lewkowich IP Day SB Ledford JR Zhou P Dienger K Wills-Karp M. Protease-activated receptor 2 activation of myeloid dendritic cells regulates allergic airway inflammation. Respir Res. (2011) 12:122. 10.1186/1465-9921-12-122 21936897 PMC3184630

[140] Kheradmand F Kiss A Xu J Lee S-H Kolattukudy PE Corry DB. A protease-activated pathway underlying Th cell type 2 activation and allergic lung disease. J Immunol. (2002) 169:5904–11. 10.4049/jimmunol.169.10.5904 12421974

[141] Park BJ Wannemuehler KA Marston BJ Govender N Pappas PG Chiller TM. Estimation of the current global burden of cryptococcal meningitis among persons living with HIV/AIDS. AIDS. (2009) 23:525–30. 10.1097/QAD.0b013e328322ffac 19182676

[142] Rajasingham R Smith RM Park BJ Jarvis JN Govender NP Chiller TM. Global burden of disease of HIV-associated cryptococcal meningitis: an updated analysis. Lancet Infect Dis. (2017) 17:873–81. 10.1016/S1473-3099(17)30243-8 28483415 PMC5818156

[143] Leopold Wager CM Wormley FL Jr. Classical versus alternative macrophage activation: the Ying and the Yang in host defense against pulmonary fungal infections. Mucosal Immunol. (2014) 7:1023. 10.1038/mi.2014.65 25073676

[144] Walsh NM Botts MR McDermott AJ Ortiz SC Wüthrich M Klein B. Infectious particle identity determines dissemination and disease outcome for the inhaled human fungal pathogen Cryptococcus. PLOS Pathog. (2019) 15:e1007777. 10.1371/journal.ppat.1007777 31247052 PMC6597114

[145] Müller U Piehler D Stenzel W Köhler G Frey O Held J. Lack of IL-4 receptor expression on T helper cells reduces T helper 2 cell polyfunctionality and confers resistance in allergic bronchopulmonary mycosis. Mucosal Immunol. (2012) 5:299. 10.1038/mi.2012.9 22333910

[146] Piehler D Eschke M Schulze B Protschka M Müller U Grahnert A. The IL-33 receptor (ST2) regulates early IL-13 production in fungus-induced allergic airway inflammation. Mucosal Immunol. (2016) 9:937–49. 10.1038/mi.2015.106 26555705

[147] Goldman DL Khine H Abadi J Lindenberg DJ Pirofski La Niang R. Serologic evidence for cryptococcus neoformans infection in early childhood. Pediatrics. (2001) 107:1–6. 10.1542/peds.107.5.e66 11331716

[148] Abadi J Pirofski L. Antibodies reactive with the cryptococcal capsular polysaccharide glucuronoxylomannan are present in sera from children with and without human immunodeficiency virus infection. J Infect Dis. (2002) 180:915–9. 10.1086/314953 10438394

[149] Goldman DL Li X Tsirilakis K Andrade C Casadevall A Vicencio AG. Increased chitinase expression and fungal-specific antibodies in the bronchoalveolar lavage fluid of asthmatic children. Clin Exp Allergy. (2012) 42:523–30. 10.1111/j.1365-2222.2011.03886.x 22092749

[150] Goldman DL Davis J Bommarito F Shao X Casadevall A. Enhanced allergic inflammation and airway responsiveness in rats with chronic cryptococcus neoformans infection: potential role for fungal pulmonary infection in the pathogenesis of asthma. J Infect Dis. (2006) 193:1178–86. 10.1086/501363 16544260

[151] Retini C Kozel TR Pietrella D Monari C Bistoni F Vecchiarelli A. Interdependency of interleukin-10 and interleukin-12 in regulation of T-cell differentiation and effector function of monocytes in response to stimulation with *Cryptococcus neoformans*. Infect Immun. (2001) 69:6064–73. 10.1128/IAI.69.10.6064-6073.2001 11553544 PMC98735

[152] Zhu Z Zheng T Homer RJ Kim YK Chen NY Cohn L. Acidic mammalian chitinase in asthmatic Th_2_ inflammation and IL-13 pathway activation. Science. (2004) 304:1678–82. 10.1126/science.1095336 15192232

[153] Van Dyken SJ Liang HE Naikawadi RP Woodruff PG Wolters PJ Erle DJ. Spontaneous chitin accumulation in airways and age-related fibrotic lung disease. Cell. (2017) 169:497–509. 10.1016/j.cell.2017.03.044 28431248 PMC5444468

[154] Vannella KM Ramalingam TR Hart KM de Queiroz Prado R Sciurba J Barron L. Acidic chitinase primes the protective immune response to gastrointestinal nematodes. Nat Immunol. (2016) 17:538–44. 10.1038/ni.3417 27043413 PMC4970463

[155] Belkaid Y Tamoutounour S. The influence of skin microorganisms on cutaneous immunity. Nat Rev Immunol. (2016) 16:353–66. 10.1038/nri.2016.48 27231051

[156] Oh J Byrd AL Deming C Conlan S Kong HH. Biogeography and individuality shape function in the human skin metagenome. Nature. (2014) 514:59. 10.1038/nature13786 25279917 PMC4185404

[157] Gupta AK Kohli Y. Prevalence of *Malassezia* species on various body sites in clinically healthy subjects representing different age group. Med Mycol. (2004) 42:35–42. 10.1080/13693780310001610056 14982112

[158] Lee YW Yim SM Lim SH Choe YB Ahn KJ. Quantitative investigation on the distribution of *Malassezia* species on healthy human skin in Korea. Mycoses. (2006) 49:405–10. 10.1111/j.1439-0507.2006.01239.x 16922793

[159] Glatz M Bosshard P Schmid-Grendelmeier P. The role of fungi in atopic dermatitis. Immunol Allergy Clin North Am. (2017) 37:63–74. 10.1016/j.iac.2016.08.012 27886911

[160] Johansson HJ Vallhov H Holm T Gehrmann U Andersson A Johansson C. Extracellular nanovesicles released from the commensal yeast *Malassezia sympodialis* are enriched in allergens and interact with cells in human skin. Sci Rep. (2018) 8:1–11. 10.1038/s41598-018-27451-9 29907748 PMC6004016

[161] Brodská P Panzner P Pizinger K Schmid-Grendelmeier P. IgE-mediated sensitization to malassezia in atopic dermatitis: More common in male patients and in head and neck type. Dermatitis. (2014) 25:120–6. 10.1097/DER.0000000000000040 24819285

[162] Svejgaard E Ølholm Larsen P Deleuran M Ternowitz T Roed-Petersen J Nilsson J. Treatment of head and neck dermatitis comparing itraconazole 200 mg and 400 mg daily for 1 week with placebo. J Eur Acad Dermatology Venereol. (2004) 18:445–9. 10.1111/j.1468-3083.2004.00963.x 15196159

[163] Yamasaki S Matsumoto M Takeuchi O Matsuzawa T Ishikawa E Sakuma M. C-type lectin Mincle is an activating receptor for pathogenic fungus, Malassezia. Proc Natl Acad Sci USA. (2009) 106:1897–902. 10.1073/pnas.0805177106 19171887 PMC2644135

[164] Ishikawa T Itoh F Yoshida S. Identification of distinct ligands for the C-type lectin receptors mincle and dectin-2 in the pathogenic fungus *Malassezia*. Cell Host Microbe. (2013) 13:477–88. 10.1016/j.chom.2013.03.008 23601109

[165] Matsuoka H Niimi A Matsumoto H Ueda T Takemura M Yamaguchi M. Specific IgE response to trichophyton and asthma severity. Chest. (2009) 135:898–903. 10.1378/chest.08-1783 19188557

[166] Ward G Jr Rose G Karlsson G Platts-Mills T Trichophyton asthma: sensitisation of bronchi and upper airways to dermatophyte antigen. Lancet. (1989) 333:859–62. 10.1016/S0140-6736(89)92863-8 2564948

[167] Fuller LC Child FC Midgley G Higgins EM. Scalp ringworm in south-east London and an analysis of a cohort of patients from a paediatric dermatology department. Br J Dermatol. (2003) 148:985–8. 10.1046/j.1365-2133.2003.05022.x 12786830

[168] Woodfolk JA Sung S-SJ Benjamin DC Lee JK Platts-Mills TAE. Distinct human T cell repertoires mediate immediate and delayed-type hypersensitivity to the *Trichophyton* antigen, Tri r 2. J Immunol. (2000) 165:4379–87. 10.4049/jimmunol.165.8.4379 11035075

[169] Fontanella S Frainay C Murray CS Simpson A Custovic A. Machine learning to identify pairwise interactions between specific IgE antibodies and their association with asthma: a cross-sectional analysis within a population-based birth cohort. PLoS Med. (2018) 15:e1002691. 10.1371/journal.pmed.1002691 30422985 PMC6233916

[170] Kneale M Bartholomew JS Davies E Denning DW. Global access to antifungal therapy and its variable cost. J Antimicrob Chemother. (2016) 71:3599–606. 10.1093/jac/dkw325 27516477

[171] Kling S Zar HJ Levin ME Green RJ Jeena PM Risenga SM. Guideline for the management of acute asthma in children: 2013 update. S Afr Med J. (2013) 103(3 Pt 3):199–207. 10.7196/SAMJ.6658 23656745

[172] Illi S von Mutius E Lau S Niggemann B Grüber C Wahn U. Perennial allergen sensitisation early in life and chronic asthma in children: a birth cohort study. Lancet. (2006) 368:763–70. 10.1016/S0140-6736(06)69286-6 16935687

[173] Gray DM Turkovic L Willemse L Visagie A Vanker A Stein DJ Gray Lung function in African infants in the Drakenstein child health study impact of lower respiratory tract illness. Am J Respir Crit Care Med. (2017) 195:212–20. 10.1164/rccm.201601-0188OC 27509359 PMC5394784

[174] Beran D Zar HJ Perrin C Menezes AM Burney P. Burden of asthma and chronic obstructive pulmonary disease and access to essential medicines in low-income and middle-income countries. Lancet Respir Med. (2015) 3:159–70. 10.1016/S2213-2600(15)00004-1 25680912

[175] Bush A. A scandal in South Africa: and not just there! Pediatr Pulmonol. (2018) 53:698–700. 10.1002/ppul.24010 29673131

